# Validation of the Moral Injury Outcome Scale in acute care nurses

**DOI:** 10.3389/fpsyt.2023.1279255

**Published:** 2023-11-29

**Authors:** Hong Tao, Jason A. Nieuwsma, Keith G. Meador, Stephanie L. Harris, Patricia S. Robinson

**Affiliations:** ^1^Center for Nursing, Whole-Person, and Academic Research, AdventHealth Research Institute, AdventHealth, Orlando, FL, United States; ^2^Integrative Mental Health, Mental Illness Research, Education and Clinical Centers MIRECC (VA), Durham, NC, United States; ^3^Department of Psychiatry and Behavioral Sciences, School of Medicine, Duke University, Durham, NC, United States; ^4^Department of Psychiatry and Behavioral Sciences, Vanderbilt University Medical Center, Nashville, TN, United States; ^5^Center for Biomedical Ethics and Society, Vanderbilt University Medical Center, Nashville, TN, United States; ^6^Graduate Department of Religion, Vanderbilt Divinity School, Nashville, TN, United States

**Keywords:** moral injury, MIOS, nurses, instrument validation, acute care, mental health

## Abstract

**Introduction:**

Moral injury, predominantly studied in military populations, has garnered increased attention in the healthcare setting, in large part due to the psychological and emotional consequences of the COVID-19 pandemic. The measurement of moral injury with instrumentation adapted from military settings and validated by frontline healthcare personnel is essential to assess prevalence and guide intervention. This study aimed to validate the Moral Injury Outcome Scale (MIOS) in the population of acute care.

**Methods:**

A sample of 309 acute care nurses completed surveys regarding moral injury, depression, anxiety, burnout, professional fulfillment, spiritual wellbeing, and post-traumatic stress disorder symptoms. Confirmatory factor analysis was conducted as well as an assessment of reliability and validity.

**Results:**

The internal consistency of the 14-item MIOS was 0.89. The scale demonstrated significant convergent and discriminant validity, and the test of construct validity confirmed the two-factor structure of shame and trust violations in this clinical population. Regression analysis indicated age, race, and marital status-related differences in the experience of moral injury.

**Discussion:**

The MIOS is valid and reliable in acute care nursing populations and demonstrates sound psychometric properties. Scores among nurses diverge from those of military personnel in areas that may inform distinctions in interventions to address moral injury in these populations.

## Introduction

Moral injury (MI) has gained prominence in the healthcare literature in recent years, principally due to the unprecedented impact of the COVID-19 pandemic. Although a universally accepted definition of MI has yet to be established, it is understood to arise from potentially morally injurious experiences that involve “perpetrating, failing to prevent, bearing witness to, or learning about acts that transgress deeply held moral beliefs and expectations” (1). Furthermore, it has been suggested that MI is a trauma-based syndrome with psychological, existential, behavioral, and interpersonal implications resulting in guilt, shame, spiritual conflict, and a loss of trust (2). Though a distinct phenomenon, MI is often associated with other mental health conditions, including post-traumatic stress disorder (PTSD), depression, anxiety, suicidality, and substance use (3–5). While initially documented in military populations, moral injury has also been noted among other professions such as police officers, veterinarians, journalists, and social services providers (6, 7).

Moral injury primarily takes place in the context of “exceptional circumstances” (8), and the COVID-19 pandemic, which precipitated difficult decision-making with scarce resources and a risk for personal harm, presented such circumstances (8, 9). Rationing of care, reallocating ventilators, and adopting a public healthcare model in lieu of a patient-centered care model likely contributed to moral injury in nurses (5, 9, 10). Shame-related moral injury may have manifested during the pandemic as a result of perceptions of the provision of suboptimal care, the inability to facilitate “good deaths”, and barriers to connecting with families due to PPE (11). Organizational and government mismanagement, unsafe staffing levels, and mixed messaging were likely contributors to trust violation-related moral injury in nurses (11–13). Research indicates the incidence of moral injury among nurses as high as approximately 40% (14). In healthcare workers on the front lines of COVID-19, moral injury has co-occurred with secondary traumatic stress, anxiety, depression, and PTSD (5, 15, 16). In addition, exposure to potentially morally injurious events (PMIEs) as a result of working during the pandemic had a significant detrimental effect on personal relationships and daily functioning (17).

With nurse staffing and retention at the forefront of priorities in healthcare and the psychological impact and work implications of the pandemic influencing nurses' intent to stay in direct care (18, 19), it is imperative to appropriately assess the psychological, emotional, and spiritual status of the nursing workforce and to design targeted interventions to address identified conditions. While instruments that measure burnout and moral distress in nurses are widely utilized (20–22), these conditions are distinct from moral injury and measure condition-specific symptoms (23–25). As proposed interventions for moral injury differ from those for related conditions (1) and often integrate a psychospiritual component (26, 27), accurately assessing moral injury in the nursing workforce and the outcomes of exposure to PMIEs is crucial.

Instruments to measure and assess moral injury were originally validated in military populations and have only recently been adapted specifically for healthcare professionals (28, Tao et al.^1^). It has been noted that some existing scales conflate the measurement of exposure to potentially morally injurious events with the outcomes of those exposures (4). Scales designed specifically to assess outcomes include the Moral Injury Symptom Scale-Military Version (MISS-MV) (29), the Expressions of Moral Injury Scale—Military Version (EMIS-M) (30), the Moral Injury and Distress Scale (MIDS) (31), and the Moral Injury Outcome Scale (MIOS) (12). To date, only the MISS—Health Professionals (MISS-HPs) has undergone validation among clinicians (28), in a sample of mixed healthcare providers, with nurses comprising 9.4% of the participants (28). However, of these measures, the MIOS has numerous advantages. It has undergone the most robust psychometric developmental process, including a three-stage process for developing items and constructing the scale (via interviews and engagement with 76 care providers and 72 service members/veterans), examining factor structure and trimming items (using five samples from four countries totaling 1,514 service members/veterans), and establishing test–retest reliability and convergent validity (12, 32). Furthermore, the MIOS contains two subscales that mirror the two widely understood potentially morally injurious experiences of both perceived betrayal and perceived transgression (33) as well as separately measuring functional outcomes as related to moral injury (34). Finally, while the MIOS was initially developed and validated for military personnel and veterans, the items on the scale are not specific to military experiences and have face validity for application in other populations. Given the evolution of moral injury as a construct initially developed and understood within military/veteran populations and now being applied in other contexts, the MIOS is a compelling measure for study and cross-validation in other samples.

This study sought to validate the MIOS in a sample of acute care nurses. Additional aims include the exploration of moral injury outcome scores in relation to documented scores from military populations and investigating associations with other potentially co-occurring conditions. This is intended to further research regarding the accurate assessment of MI and related factors among healthcare personnel, which may inform interventions that can address the wellbeing of direct care providers.

## Methods

This cross-sectional survey was conducted from June 2022 to February 2023 in central Florida. It was approved by the hospital's institutional review board. Electronic informed consent was obtained from the participants.

### Participants and setting

To recruit nurses, an invitation email with survey information, a QR code, and a flier was sent to potential participants. The inclusion criteria include: a. currently working as a registered nurse (e.g., LPN, RN, assistant nurse manager, nurse manager, and nurse educator) at identified hospitals within the state of Florida, b. access to a computer or mobile device to complete the survey, and c. ability to read and understand English. The survey was conducted on Qualtrics, a HIPAA-compliant, web-based survey platform. Each participant was offered a $30 stipend upon the completion of the survey. In addition to MIOS and MI functional outcomes, the survey package includes a demographic questionnaire (age, sex, marital status, race, religion, ethnicity, educational level, workplace, years of working experience, and years of current role) and other scales as described below, including moral injury, depression, anxiety, spiritual wellbeing, professional fulfillment, burnout, and PTSD scales.

### Measures

#### Moral injury outcomes scale

The Moral Injury Outcomes Scale (MIOS) was developed and validated initially and independently by a multi-disciplinary consortium (12), with particular attention to the engagement of an international sample of active-duty service members, veterans, clinicians, and chaplains to establish content validity (35, 36). Introductory questions probe exposure to three types of potentially morally injurious events and use this exposure or another current distressing event as a frame of reference for response to the survey instrument. The MIOS incorporates 14 items that comprise two subscales: shame-related and trust violation-related. Shame-related moral injury is associated with self-facing concerns about being negatively perceived by valued others (1, 2, 6). Trust violation-related moral injury is externally facing and involves feelings of betrayal, most often involving those in positions of power (1, 2, 6). To further evaluate moral injury-related outcomes, the authors also integrated the 7-item Brief Inventory of Psychosocial Functioning (B-IPF) (34) to measure the impact on social relationships, work, and daily activities (36).

#### Expressions of moral injury scale

The Expressions of Moral Injury Scale (EMIS) (30) is a valid and reliable measure of expressions of MI in two factors: self-directed MI and other-directed MI. The 10-item self-directed moral injury subscale items were used for this study. All items were rated on a Likert scale from 1 (strongly disagree) to 5 (strongly agree), with higher scores indicating greater levels of current moral injury. The Cronbach's α for this study 0.89.

#### Moral injury event scale

The Moral Injury Event Scale (MIES) (37) is comprised of nine statements with two factors: perceived transgressions by self of other and perceived betrayal by others. The responses range from 1 (strongly agree) to 6 (strongly disagree), with higher scores indicating greater moral injury. The Cronbach's α for this study is 0.88 for perceived transgressions by self of others and 0.82 for perceived betrayal by others.

#### Patient health questionnaire-9

The 9-item Patient Health Questionnaire-9 (PHQ-9) (38) has been widely used as a self-administered screening tool for assessing the severity of depressive symptoms. Each item of PHQ-9 was scored on a scale of 0–3 (0 = not at all; 1 = several days; 2 = more than a week; 3 = nearly every day). The PHQ-9 total score ranges from 0 to 27 (scores of 5–9 are classified as mild depression; 10–14 as moderate depression; 15–19 as moderately severe depression; ≥20 as severe depression). The Cronbach's α for this study is 0.90.

#### Generalized anxiety disorder-7

The Generalized Anxiety Disorder-7 (GAD-7) (39) is a valid and reliable 7-item screening tool for generalized anxiety disorder over the previous 2 weeks. Each item of GAD-7 is scored on a scale of 0–3 (0 = not at all; 1 = several days; 2 = more than the days; 3 = nearly every day). The following cutoffs correlate with the level of anxiety severity: scores 0–4: minimal anxiety; scores 5–9: mild anxiety; scores 10–14: moderate anxiety; and score < 15: severe anxiety. The Cronbach's α for this study is 0.93.

#### Maslach burnout inventory

Maslach Burnout Inventory (MBI) (40) is composed of 22 items with three factors: emotional exhaustion, depersonalization, and personal accomplishment. Items are rated on a 1–7 scale from “never” to “every day”, with positively worded items reverse-scored. The Cronbach's α for this study is 0.94, 0.76, and 0.80 for the three subscales: emotional exhaustion, depersonalization, and professional accomplishment, respectively.

#### Stanford professional fulfillment index

The Stanford Professional Fulfillment (SPF) (41) consists of 16 items with three subscales: a 6-item professional fulfillment subscale; a 4-item work exhaustion subscale; and a 6-item interpersonal disengagement subscale. Items are rated from 0 to 4, with higher professional fulfillment and lower work exhaustion and interpersonal disengagement scores representing more favorable responses. The Cronbach's α for this study is 0.91, 0.90, and 0.93 for the three subscales: professional fulfillment, work exhaustion, and interpersonal disengagement.

#### Functional assessment of chronic illness therapy-spiritual wellbeing—Non-illness

The Functional Assessment of Chronic Illness Therapy—Spiritual Wellbeing—Non-Illness (FACIT-Sp) used in this study is composed of 12 items using a 5-point Likert-type scale (0 = not at all; 1 = a little bit; 2 = somewhat; 3 = quite a bit; and 4 = very much). Higher scores indicate better spiritual wellbeing. The Cronbach's α for this study is 0.92.

#### Post-traumatic stress disorder checklist (PCL-5)

The Post-traumatic stress disorder (PCL-5) (42) is a 20-item self-report measure that assesses the presence and severity of PTSD symptoms. Item scores range from 0 (not at all) to 4 (extremely), with higher scores indicating severer symptoms. The Cronbach's α for this study is 0.96.

### Statistical analyses

Data analysis was performed using SPSS 28 software (IBM SPSS Statistics for Windows, version 25.0, IBM Corp., Armonk, NY, USA). Continuous variables were expressed as mean ± standard deviation; categorical data were summarized as frequencies and percentages. Cronbach's alpha was used to assess the internal consistency of the MIOS. A confirmatory factor analysis (CFA) using the statistical package analysis of moment structures (AMOS 28) was conducted to confirm the structure of the MIOS. Absolute fit indices were calculated to evaluate the model fit (43), including normed chi-square (X^2^/df), root mean square error of approximation (RMESA), and goodness-of-fit statistic (GFI). To test the convergent, Pearson's correlation coefficient was adopted. In addition, a one-way ANOVA was used to compare the moral injury scores among groups, and linear regression was used to identify the factors relevant to moral injury in RNs.

## Results

### Characteristics of the sample

A total of 309 nurses completed the survey and were included in the data analysis. The demographic characteristics of the sample are presented in Table 1. Most respondents were female (91.1%), white (71.5%), non-Hispanic/Latino (87.0%), Christian (85.7%), and had a bachelor's or higher degree (74.0%). More than half (54.4%) were between 31 and 49 years old. More than half of the respondents were married. Work settings included acute care inpatient units (43.7%), COVID-19 units (0.6%), intensive care (18.1%), emergency departments (6.8%), or others (22.0%). Those who selected other work settings were prompted to specify their work area, and participants identified units throughout the hospital, including mother and infant care (labor and delivery, mother/baby), procedural areas (vascular access, interventional radiology, and cath lab), and other inpatient units (progressive care and med-surg). About half of the participants (49.5%) had 11 or more years of working experience.

**Table 1:** Demographic characteristics for participants (*n* = 309).

**Participant's Characteristics**		***n***	**%**
Sex	Female	278	91.1
	Male	27	8.9
Age	18–24	10	3.2
	25–30	53	17.2
	31–39	85	27.5
	40–49	83	26.9
	50–59	53	17.2
	60+	25	8.1
Marital status	Divorced/Separated	37	12.0
	Married	191	61.8
	Partnered	14	4.5
	Single	62	20.1
	Widowed	5	1.6
Race	Asian	22	7.1
	Black or African-American	40	12.9
	Multiracial	14	4.5
	Native Hawaiian or Pacific Islander	1	0.3
	Other	11	3.6
	White	221	71.5
Religion	Buddhist	1	0.3
	Christian	264	85.7
	Hindu	6	1.9
	Jewish	1	0.3
	Muslim	2	0.6
	Other	34	11.0
Ethnicity	Hispanic/Latino	40	13.0
	Non-Hispanic/Non-Latino	268	87.0
Educational level	Associate's degree	67	21.8
	Bachelor's degree	181	59.0
	Doctoral degree	5	1.6
	Master's degree	41	13.4
	Nursing diploma	13	4.2
Workplace	Acute care inpatient units	135	43.7
	COVID-19 unit	2	0.6
	Emergency	21	6.8
	Intensive care	56	18.1
	Other	68	22.0
	Perioperative	27	8.7
Years of experience	< 1	11	3.6
	1–2	23	7.5
	3–5	52	16.9
	6–10	69	22.5
	11+	152	49.5
Years of role	< 1	48	15.5
	1–2	87	28.2
	3–5	87	28.2
	6–10	45	14.6
	11+	42	13.6

### Reliability

Reliability is the degree to which an instrument consistently measures a construct. A Cronbach's alpha analysis was used to assess the modified MIOS internal consistency with 14 items and the 2 subscales (shame-related outcomes and trust violation-related outcomes) with 7 items for each subscale. The internal consistency of the 14 items, as measured by Cronbach's alpha, was 0.89, 0.86, and 0.80 for the two subscales (Table 2).

**Table 2:** Reliability of the MIOS for RN (with items removed and total score).

**Item**	**MIOS (*****n*** = **309)**		**Shame**		**Trust violation**	
	**Mean (SD)**	**Cronbach's alpha if removed**	**Mean (SD)**	**Cronbach's alpha if removed**	**Mean (SD)**	**Cronbach's alpha if removed**
MIOS1	1.34 (1.14)	0.89	1.34 (1.14)	0.87		
MIOS2	1.37 (1.10)	0.88			1.37 (1.10)	0.76
MIOS3	0.69 (0.85)	0.88	0.69 (0.85)	0.83		
MIOS4	0.99 (0.97)	0.88			0.99 (0.97)	0.76
MIOS5	0.66 (0.71)	0.89			0.66 (0.71)	0.78
MIOS6	2.17 (1.22)	0.89			2.17 (1.21)	0.80
MIOS7	0.52 (0.72)	0.88	0.52 (0.72)	0.84		
MIOS8	0.97 (1.06)	0.88	0.97 (1.06)	0.85		
MIOS9	0.60 (0.88)	0.89			0.60 (0.88)	0.81
MIOS10	1.45 (1.15)	0.88			1.45 (1.15)	0.73
MIOS11	0.83 (0.93)	0.88			0.83 (0.93)	0.76
MIOS12	0.70 (0.85)	0.88	0.70 (0.85)	0.83		
MIOS13	0.83 (0.94)	0.88	0.83 (0.94)	0.83		
MIOS14	0.74 (0.89)	0.88	0.74 (0.89)	0.84		
Total	13.86 (8.71)	0.89	5.81 (4.80)	0.86	8.06 (4.74)	0.80

### Convergent validity

As evidence for convergent validity, Table 3 depicts significant positive correlations between the MIOS, the two subscales, and MIOS functional outcomes with other scales/subscales that measure similar concepts. These measures were significantly positively related to self-directed moral injury, perceived transgressions by self of others, perceived betrayal by others, depression, anxiety, emotional exhaustion, depersonalization, work exhaustion, interpersonal disengagement, and post-traumatic stress disorder. They were significantly negatively related to perceived achievement, professional fulfillment, and spiritual wellbeing.

**Table 3:** Correlation between the moral injury, mental health, wellbeing, and burnout for nurses.

	**MIOS**	**Shame**	**Trust violation**	**MIOS functional outcomes**
EMIS (self-directed MI)	0.642^**^	0.618^**^	0.543^**^	0.509^**^
MIES (perceived transgressions by self of others)	0.410^**^	0.345^**^	0.400^**^	0.268^**^
MIES (perceived betrayal by others)	0.514^**^	0.378^**^	0.551^**^	0.383^**^
PHQ	0.522^**^	0.443^**^	0.505^**^	0.516^**^
GAD	0.501^**^	0.410^**^	0.501^**^	0.463^**^
MBI (emotional exhaustion)	0.549^**^	0.445^**^	0.555^**^	0.422^**^
MBI (depersonalization)	0.474^**^	0.396^**^	0.463^**^	0.329^**^
MBI (perceived achievement)	−0.403^**^	−0.367^**^	−0.358^**^	−0.244^**^
SPF (professional fulfillment)	−0.524^**^	−0.435^**^	−0.521^**^	−0.422^**^
SPF (work exhaustion)	0.477^**^	0.363^**^	0.507^**^	0.376^**^
SPF (interpersonal disengagement)	0.568^**^	0.464^**^	0.563^**^	0.423^**^
FACIT (spiritual wellbeing)	−0.634^**^	−0.547^**^	−0.617^**^	−0.504^**^
PCL (PTSD)	0.629^**^	0.515^**^	0.626^**^	0.549^**^

** *p* < 0.001.

### Construct validity

**Figure 1:**
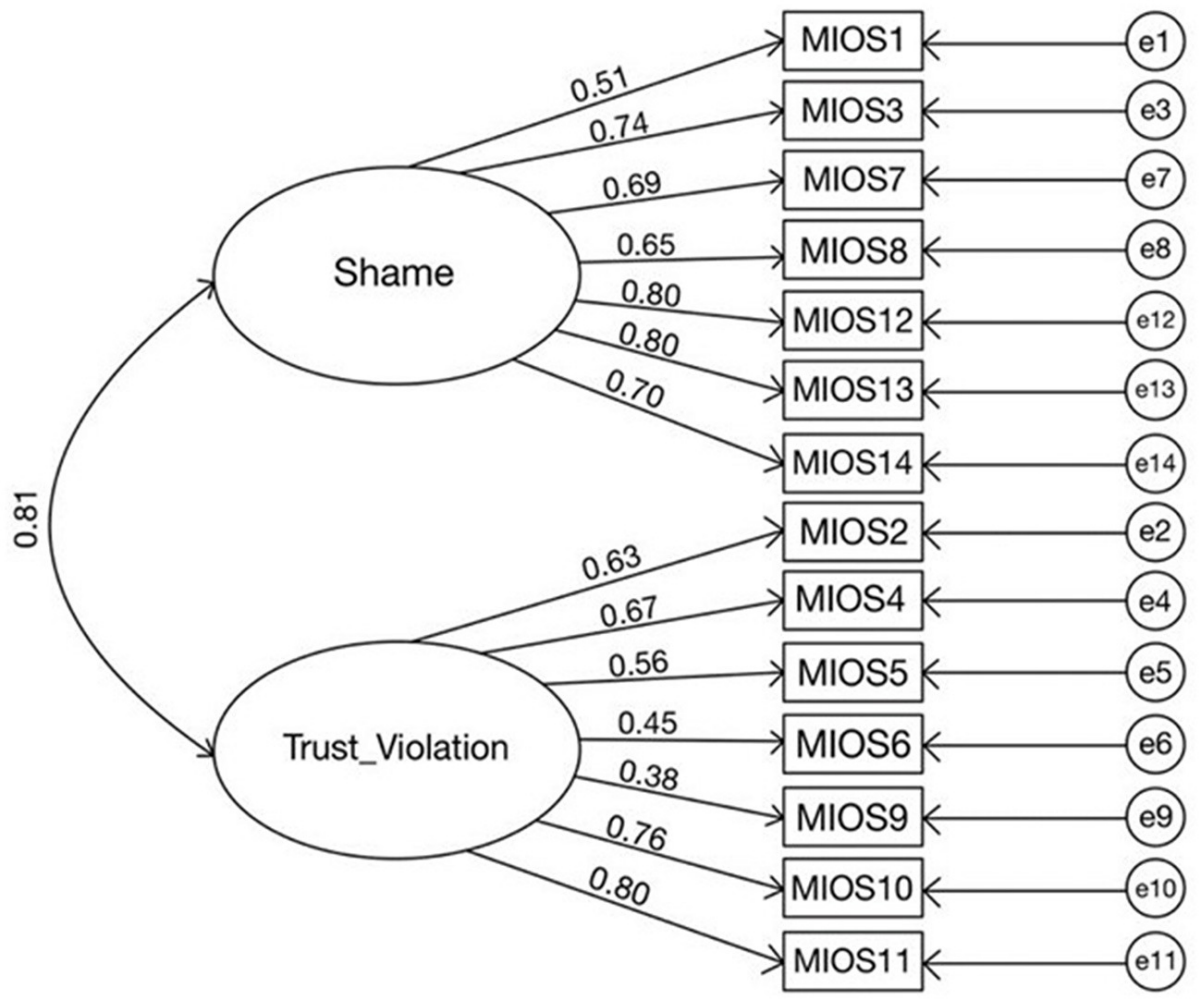
Structural model with standardized path coefficients.

Confirmatory factor analysis (CFA) using the statistical package analysis of moment structures (AMOS 28) was conducted to verify the two-factor structure of the MIOS. Figure 1 shows the results with X^2^/df = 3.019 < 5.0 (X^2^ = 229.48, df = 76) RMSEA = 0.08, GFI = 0.092 and CFI = 0.915, which is acceptable (41).

### Comparison of moral injury between demographic groups

The outcome variables were compared between demographic groups, and the results are displayed in Table 4. Based on categorical data collected regarding age and years of experience in nursing, nurses were categorized *a priori* into older (≥40 years) and more experienced (≥11 years of nursing experience). Nurses who were below 40 scored significantly higher on the MIOS (*p* < 0.01), shame-related subscale (*p* < 0.05), and trust violation-related subscale (*p* < 0.05) than those 40 and above. Compared to those with 11 or more years of working experience, those with <11 years of working experience scored higher on the MIOS and the shame-related subscale (*p* < 0.05). Married/partnered nurses had less functional impairment on the B-IPF than those who were divorced, separated, singled, or widowed (*p* < 0.05). Non-white participants had lower scores on the MIOS (*p* < 0.05) and trust violation-related subscale (*p* < 0.05) than white participants. Hispanics/Latinos scored lower on the trust violation-related subscale (*p* < 0.05) compared to non-Hispanics/Non-Latinos.

**Table 4:** Comparison of moral injury between demographic groups of nurses.

		**MIOS**	**Shame**	**Trust violation**	**Functional outcomes (*n* does not apply to this column)**
Total	N (309)	13.86 (8.71)	5.81 (4.80)	8.06 (4.74)	25.92 (15.76)
	Group (n)	Mean (SD)	Mean (SD)	Mean (SD)	Mean (SD)
Age	Below 40 (148)	15.22 (9.28)^**^	6.51 (5.10)^*^	8.70 (5.04)^*^	26.79 (16.36)
	40 and above (161)	12.61 (7.98)	5.16 (4.43)	7.46 (4.36)	25.08 (15.17)
Gender	Female (278)	13.64 (8.58)	5.59 (4.70)	8.00 (5.31)	26.15 (15.60)
	Male (27)	15.41 (8.88)	7.41 (4.81)	8.04 (4.72)	23.22 (17.13)
Marital status	Divorced/Separated/Single/Widowed (104)	14.38 (8.33)	5.76 (4.59)	8.63 (4.68)	28.54 (15.54)^*^
	Married/Partnered (209)	13.60 (8.90)	5.83 (4.91)	7.77 (4.76)	24.54 (15.74)
Race	Non-White (88)	12.19 (8.0)^*^	5.09 (4.26)	7.10 (4.58)^*^	27.95 (17.90)
	White (221)	14.52 (8.9)	6.09 (4.98)	8.43 (4.76)	25.12 (14.80)
Ethnicity	Hispanic/Latino (40)	11.70 (7.68)	5.10 (3.86)	6.60 (4.49)^*^	27.74 (18.66)
	Non-Hispanic/Non-Latino (268)	14.18 (8.37)	5.93 (4.92)	8.25 (4.74)	25.66 (15.31)
Educational level	Nursing diploma/Associate degree (80)	13.36 (8.67)	5.34 (4.90)	8.03 (4.80)	26.66 (16.61)
	Bachelor's degree (181)	13.93 (8.51)	5.82 (4.73)	8.11 (4.53)	25.91 (15.85)
Master's/Doctoral degree (46)	14.93 (9.46)	6.72 (4.92)	8.22 (5.35)	25.46 (14.15)
Workplace	Others (230)	13.46 (8.78)	5.63 (4.74)	7.83 (4.80)	26.02 (15.77)
	ED/ICU/COVID-19 Unit (79)	15.04 (8.46)	6.33 (4.96)	8.71 (4.52)	25.65 (15.85)
Years of experience	< 11 years (155)	14.99 (9.28)^*^	6.46 (5.19)^*^	8.53 (4.87)	27.29 (15.92)
	11 or more years (152)	12.74 (7.99)	5.14 (4.31)	7.60 (4.56)	24.61 (15.59)
Years in role	< 3 years (135)	14.10 (9.00)	5.99 (4.93)	8.10 (4.84)	26.87 (15.34)
	3 or more years (174)	13.68 (8.50)	5.66 (4.71)	8.02 (4/68)	25.18 (16.09)

* *p* < 0.05;

** *p* < 0.01.

### Factors associated with MI

To examine the factors associated with MI, linear regressions were applied, including all the significant variables (non-significant values were excluded) in Table 4 as independent variables and each of the outcome variables as dependent variables. Table 5 shows the results from the linear regressions. Nurses aged 40 and above tended to have lower moral injury and trust violation-related outcomes. White nurses tended to have higher moral injury (MIOS) scores and trust violation-related outcomes. Being married/partnered is associated with lower functional outcome scores.

**Table 5:** Linear regression.

**Dependent variable**	**Nurses**				
	**Independent variable**	**B**	**Beta**	***t***	***p***
MIOS	Age 40 and above	−3.095	−0.178	−3.129	0.002
	White	2.964	0.154	2.707	0.007
Trust violation	Age 40 and above	−1.517	−0.160	−2.811	0.005
	White	1.642	0.157	2.749	0.006
Functional outcome score	Married/partnered	−4.001	−0.121	−2.098	0.037

## Discussion

The potential for moral injury among healthcare workers has been increasingly recognized in recent years, particularly as a result of the COVID-19 pandemic. However, most measures of moral injury have been developed and validated in samples of military veterans. As a consequence, there is a need to adapt and validate these measures for application in populations of healthcare workers, including nurses, who were especially impacted during the pandemic. As one of the most robustly validated measures of moral injury to date, the MIOS was selected for validation among a sample of acute care nurses.

Overall, the MIOS demonstrated good psychometric properties in the study sample. A confirmatory factory analysis demonstrated an acceptable model fit for the two-factor structure of the MIOS, mapping to the shame-related and trust violation-related subscales. Reliability analyses indicated good internal consistency for the overall MIOS and acceptable internal consistency for the two subscales. Convergent validity was demonstrated by significant correlations between higher MIOS scores and higher scores on measures of depression, anxiety, burnout, and post-traumatic stress. These findings all generally align with previous research validating MIOS in military populations.

Findings from the present study focused on the relationship between moral injury and participant demographics are consistent with previous research that found lower moral injury among older individuals (44) and those with social support (45), but contradict prior research that found higher rates of moral injury among non-white individuals (16). This last finding merits further investigation in future research, including the extent to which these contradictory findings from different studies might be explained by differences in other demographic variables (e.g., age and years of experience), mental health, and cultural factors.

In comparing scores on the MIOS between the current study with acute care nurses and the initial validation study with military veterans (36), the veteran sample produced higher average scores on all 14 MIOS items. This parallels other research that has found military veterans to have higher scores on measures of moral injury in comparison to healthcare workers (16). Within both samples, the MIOS items most regularly endorsed reflect feelings of disgust and loss of faith in others. These findings suggest that the contours of moral injury may be similar between these two populations, while at the same time, the differences in the prevalence of endorsement should caution against drawing an overly reductionistic parallel between the two contexts. There are meaningful differences between military personnel and healthcare providers along many dimensions, including missions, power dynamics, lethality decisions, personal autonomy, and social norms and surroundings. These differences are important to account for in designing and providing care to people with moral injuries. Simultaneously, the present study indicates that the MIOS has acceptable psychometric properties for use among nurses and potentially other healthcare providers.

More studies are needed to determine whether and how to make use of the MIOS within organizational healthcare contexts. Both the present study and prior research looking at the MIOS in veterans have examined the scale's psychometric properties, but efforts remain underway to assess the measure's utility in clinical contexts. The MIOS is a promising tool both for identifying moral injury as well as for tracking change across time. However, no published studies have yet examined its use for either of these purposes.

Within healthcare, the MIOS could prove a useful tool for helping to clarify whether a provider's distress is due to moral injury or to another cause, such as burnout or mental health struggles. Such differentiation may help indicate what type of intervention is most needed. For instance, an individual experiencing both moral injury and burnout may benefit from attention specifically directed to the morally injurious experience(s), whereas someone experiencing burnout may benefit from more general care focused on things such as coping and distress tolerance. The MIOS could also be useful at a broader healthcare system level. For instance, higher levels of moral injury across an entire department may point to systemic problems that need addressing.

Despite the strengths of this study, including a relatively large sample size and a comprehensive assessment of constructs related to moral injury, there are also limitations to consider. The sample consists predominantly of acute care nurses from central Florida, potentially limiting geographic generalizability. Future research should seek to replicate findings in other and broader samples. Furthermore, it is important to recognize that data were collected in 2022–2023, a time during which the pandemic was still ongoing but past its peak. Some of the newest nurses in the sample may not have practiced during the height of the pandemic. Finally, as with much research on moral injury, this was a cross-sectional study, and longitudinal research is needed to explore the dynamic nature of moral injury and its effects more optimally over time.

The validation of the MIOS in this sample of acute care nurses demonstrates that it can be a useful measure of potential moral injury in this population. The findings contribute to the growing body of literature on moral injury among healthcare workers and emphasize the need for tailored interventions. By accurately identifying and assessing moral injury, healthcare organizations can work to develop targeted strategies to reduce the potential for moral injury in healthcare workers, to support their psychosocial-spiritual wellbeing, and to promote their retention in direct care roles.

## Data availability statement

The original contributions presented in the study are included in the article/supplementary material, further inquiries can be directed to the corresponding author.

## Ethics statement

The studies involving humans were approved by AdventHealth Institutional Review Board Study ID 1497904. The studies were conducted in accordance with the local legislation and institutional requirements. The participants provided their written informed consent to participate in this study.

## Author contributions

HT: Formal analysis, Investigation, Methodology, Writing—original draft, Writing—review & editing. JN: Conceptualization, Writing—original draft, Writing—review & editing. KM: Conceptualization, Writing—original draft, Writing—review & editing. SH: Data curation, Project administration, Writing—original draft, Writing—review & editing. PR: Conceptualization, Investigation, Methodology, Supervision, Writing—review & editing.

## Footnotes

^1^Tao H, Anderson G, Harris S, Sawyer A, Bailey A, Robinson P. *Development and validation of the Moral Injury Symptom Scale - Clinician Version - Short Form (MISS-CV-SF) Under Review*.

## References

[1] Litz BT Stein N Delaney E Lebowitz L Nash WP Silva C. Moral injury and moral repair in war veterans: a preliminary model and intervention strategy. Clin Psychol Rev. (2009) 29:695–706. 10.1016/j.cpr.2009.07.003 19683376

[2] Jinkerson JD. Defining and assessing moral injury: a syndrome perspective. Traumatology (Tallahassee, Fla). (2016) 22:122–30. 10.1037/trm0000069

[3] Hall NA Everson AT Billingsley MR Miller MB. Moral injury, mental health and behavioural health outcomes: a systematic review of the literature. Clin Psychol Psychother. (2022) 29:92–110. 10.1002/cpp.2607 33931926

[4] McEwen C Alisic E Jobson L. Moral injury and mental health: a systematic review and meta-analysis. Traumatology (Tallahassee, Fla). (2021) 27:303–15. 10.1037/trm0000287

[5] Amsalem D Lazarov A Markowitz JC Naiman A Smith TE Dixon LB. Psychiatric symptoms and moral injury among us healthcare workers in the Covid-19 era. BMC Psychiatry. (2021) 21:546. 10.1186/s12888-021-03565-9 34740357 PMC8571005

[6] Griffin BJ Purcell N Burkman K Litz BT Bryan CJ Schmitz M. Moral injury: An integrative review. J Trauma Stress. (2019) 32:350–62. 10.1002/jts.22362 30688367

[7] Williamson V Stevelink SAM Greenberg N. Occupational moral injury and mental health: Systematic review and meta-analysis. Br J Psychiatry. (2018) 212:339–46. 10.1192/bjp.2018.55 29786495

[8] Cartolovni A Stolt M Scott PA Suhonen R. Moral injury in healthcare professionals: A scoping review and discussion. Nurs Ethics. (2021) 28:590–602. 10.1177/0969733020966776 33427020 PMC8366182

[9] Lesley M. Psychoanalytic perspectives on moral injury in nurses on the frontlines of the Covid-19 pandemic. J Am Psychiatr Nurses Assoc. (2021) 27:72–6. 10.1177/1078390320960535 32951499

[10] Hossain F Clatty A. Self-care strategies in response to nurses' moral injury during Covid-19 pandemic. Nurs Ethics. (2021) 28:23–32. 10.1177/0969733020961825 33124492 PMC7604672

[11] Xue Y Lopes J Ritchie K D'Alessandro AM Banfield L McCabe RE. Potential circumstances associated with moral injury and moral distress in healthcare workers and public safety personnel across the globe during Covid-19: a scoping review. Front Psychiatry. (2022) 13:863232. 10.3389/fpsyt.2022.863232 35770054 PMC9234401

[12] Hegarty S Lamb D Stevelink SA Bhundia R Raine R Doherty MJ. ‘It hurts your heart': Frontline healthcare worker experiences of moral injury during the Covid-19 pandemic. Eur J Psychotraumatol. (2022) 13:2128028. 10.1080/20008066.2022.2128028 36276556 PMC9586685

[13] Nelson KE Hanson GC Boyce D Ley CD Swavely D Reina M. Organizational impact on healthcare workers' moral injury during Covid-19: a mixed-methods analysis. J Nurs Adm (2022) 52:57. 10.1097/NNA.0000000000001103 34910709 PMC9199451

[14] Rushton CH Thomas TA Antonsdottir IM Nelson KE Boyce D Vioral A. Moral injury and moral resilience in health care workers during Covid-19 pandemic. J Palliat Med. (2022) 25:712–9. 10.1089/jpm.2021.0076 34678091 PMC9081047

[15] Litam SDA Balkin RS. Moral injury in health-care workers during Covid-19 pandemic. Traumatology. (2021) 27:14. 10.1037/trm0000290

[16] Nieuwsma JA O'Brien EC Xu H Smigelsky MA Meador KG. Patterns of potential moral injury in post-9/11 combat veterans and Covid-19 healthcare workers. J Gen Intern Med. (2022) 37:2033–40. 10.1007/s11606-022-07487-4 35381899 PMC8982664

[17] Borges LM Holliday R Barnes SM Bahraini NH Kinney A Forster JE. A longitudinal analysis of the role of potentially morally injurious events on Covid-19-related psychosocial functioning among healthcare providers. PLoS ONE. (2021) 16:e0260033. 10.1371/journal.pone.0260033 34767617 PMC8589198

[18] Chen H-M Liu C-C Yang S-Y Wang Y-R Hsieh P-L. Factors related to care competence, workplace stress, and intention to stay among novice nurses during the coronavirus disease (Covid-19) pandemic. Int J Environ Res Public Health. (2021) 18:2122. 10.3390/ijerph18042122 33671613 PMC7926418

[19] Raso R Fitzpatrick JJ Masick K. Nurses' intent to leave their position and the profession during the Covid-19 pandemic. J Nurs Adm. (2021) 51:488–94. 10.1097/NNA.0000000000001052 34519700

[20] Corley MC Elswick RK Gorman M Clor T. Development and evaluation of a moral distress scale. J Adv Nurs. (2001) 33:250–6. 10.1111/j.1365-2648.2001.01658.x 11168709

[21] Schneider A Forster JE Mealer M. Exploratory and confirmatory factor analysis of the maslach burnout inventory to measure burnout syndrome in critical care nurses. J Nurs Meas. (2020) 28:E18–29. 10.1891/JNM-D-18-00055 32179723

[22] Leiter MP Maslach C. Latent burnout profiles: a new approach to understanding the burnout experience. Burn Res. (2016) 3:89–100. 10.1016/j.burn.2016.09.001

[23] Dean W Talbot S Dean A. Reframing clinician distress: moral injury not burnout. Federal Practitioner. (2019) 36:400. 31571807 PMC6752815

[24] Musto LC Rodney PA Vanderheide R. Toward interventions to address moral distress: navigating structure and agency. Nurs Ethics. (2015) 22:91–102. 10.1177/0969733014534879 24917268

[25] Dale LP Cuffe SP Sambuco N Guastello AD Leon KG Nunez LV. Morally distressing experiences, moral injury, and burnout in Florida healthcare providers during the Covid-19 pandemic. Int J Environmen Res Public Health. (2021) 18:12319. 10.3390/ijerph182312319 34886045 PMC8656473

[26] Harris JI Chamberlin ES Engdahl B Ayre A Usset T Mendez D. Spiritually integrated interventions for ptsd and moral injury: a review. Curr Treat Options Psychiatry. (2021) 8:196–212. 10.1007/s40501-021-00248-w

[27] Wortmann JH Nieuwsma JA King HA Fernandez P Jackson GL Smigelsky MA. Collaborative spiritual care for moral injury in the veterans affairs healthcare system (va): Results from a national survey of va chaplains. J Health Care Chaplain. (2022) 28:S9–S24. 10.1080/08854726.2021.2004847 34825859

[28] Mantri S Lawson JM Wang Z Koenig HG. Identifying moral injury in healthcare professionals: the moral injury symptom scale-hp. J Relig Health. (2020) 59:2323–40. 10.1007/s10943-020-01065-w 32681398 PMC7366883

[29] Koenig HG Ames D Youssef NA Oliver JP Volk F Teng EJ. The moral injury symptom scale-military version. J Relig Health. (2018) 57:249–65. 10.1007/s10943-017-0531-9 29196962

[30] Currier JM Farnsworth JK Drescher KD McDermott RC Sims BM Albright DL. Development and evaluation of the expressions of moral injury scale-military version. Clin Psychol Psychother. (2018) 25:474–88. 10.1002/cpp.2170 29282787

[31] Norman SB Griffin BJ Pietrzak RH McLean C Hamblen JL Maguen S. The moral injury and distress scale: psychometric evaluation and initial validation in three high-risk populations. Psychol Trauma. (2023). 10.1037/tra0001533 37347882

[32] Levine SZ Laufer A Stein E Hamama-Raz Y Solomon Z. Examining the relationship between resilience and posttraumatic growth. J Trauma Stress (2009) 22:282–6. 10.1002/jts.20409 19593805

[33] Shay J. Moral injury. Psychoanal Psychol. (2014) 31:182. 10.1037/a0036090

[34] Kleiman SE Bovin MJ Black SK Rodriguez P Brown LG Brown ME. Psychometric properties of a brief measure of posttraumatic stress disorder–related impairment: The brief inventory of psychosocial functioning. Psychol Serv. (2020) 17:187. 10.1037/ser0000306 30299150

[35] Yeterian JD Berke DS Carney JR McIntyre-Smith A St Cyr K King L. Defining and measuring moral injury: Rationale, design, and preliminary findings from the moral injury outcome scale consortium. J Trauma Stress. (2019) 32:363–72. 10.1002/jts.22380 30947372

[36] Litz BT Plouffe RA Nazarov A Murphy D Phelps A Coady A. Defining and assessing the syndrome of moral injury: initial findings of the moral injury outcome scale consortium. Front Psychiat. (2022) 13:923928. 10.3389/fpsyt.2022.923928 35873252 PMC9297368

[37] Nash WP Marino Carper TL Mills MA Au T Goldsmith A Litz BT. Psychometric evaluation of the moral injury events scale. Mil Med. (2013) 178:646–52. 10.7205/MILMED-D-13-00017 23756071

[38] Kroenke K Spitzer RL Williams JB. The phq-9: Validity of a brief depression severity measure. J Gen Intern Med. (2001) 16:606–13. 10.1046/j.1525-1497.2001.016009606.x 11556941 PMC1495268

[39] Spitzer RL Kroenke K Williams JB Löwe B A. brief measure for assessing generalized anxiety disorder: the gad-7. Arch Intern Med. (2006) 166:1092–7. 10.1001/archinte.166.10.1092 16717171

[40] Maslach C Jackson S Leiter M. Maslach Burnout Inventory Manual 3rd ed. Palo Alto, CA: Consulting Psychologists Press. (1996).

[41] Trockel M Bohman B Lesure E Hamidi MS Welle D Roberts L. A brief instrument to assess both burnout and professional fulfillment in physicians: Reliability and validity, including correlation with self-reported medical errors, in a sample of resident and practicing physicians. Acad Psychiatry. (2018) 42:11–24. 10.1007/s40596-017-0849-3 29196982 PMC5794850

[42] Blevins CA Weathers FW Davis MT Witte TK Domino JL. The posttraumatic stress disorder checklist for dsm-5 (pcl-5): development and initial psychometric evaluation. J Trauma Stress. (2015) 28:489–98. 10.1002/jts.22059 26606250

[43] McDonald RP Ho M-HR. Principles and practice in reporting structural equation analyses. Psycholl Methods. (2002) 7:64. 10.1037/1082-989X.7.1.64 11928891

[44] Riedel PL Kreh A Kulcar V Lieber A Juen B. A scoping review of moral stressors, moral distress and moral injury in healthcare workers during Covid-19. Int J Environ Res Public Health. (2022) 19:1666. 10.3390/ijerph19031666 35162689 PMC8835282

[45] D'Alessandro AM Ritchie K McCabe RE Lanius RA Heber A Smith P. Healthcare workers and Covid-19-related moral injury: an interpersonally-focused approach informed by ptsd. Front Psychiatry. (2022) 12:784523. 10.3389/fpsyt.2021.784523 35264983 PMC8900218

